# *HOXA1* 3′UTR Methylation Is a Potential Prognostic Biomarker in Oral Squamous cell Carcinoma

**DOI:** 10.3390/cancers16050874

**Published:** 2024-02-22

**Authors:** Bruna Pereira Sorroche, Keila Cristina Miranda, Caroline Moraes Beltrami, Lidia Maria Rebolho Batista Arantes, Luiz Paulo Kowalski, Fabio Albuquerque Marchi, Silvia Regina Rogatto, Janete Dias Almeida

**Affiliations:** 1 Molecular Oncology Research Center, Barretos Cancer Hospital, Barretos 14784-400, Brazil; bruna.sorroche20@edu.hospitaldeamor.com.br (B.P.S.); lidia.arantes@hospitaldeamor.com.br (L.M.R.B.A.); 2 Department of Biosciences and Oral Diagnosis, Institute of Science and Technology, São Paulo State University (UNESP), São José dos Campos 12224-300, Brazil; keilamiranda94@gmail.com; 3 International Research Center, A.C. Camargo Cancer Center, São Paulo 01508-010, Brazil; cahbeltrami@gmail.com; 4 Head and Neck Surgery and Otorhinolaryngology Department, AC Camargo Cancer Center, Latin American Cooperative Oncology Group, São Paulo 01509-010, Brazil; lpkowalski@accamargo.org.br; 5 Head and Neck Surgery Department and LIM 28, University of São Paulo Medical School, São Paulo 01246-903, Brazil; 6 Center for Translational Research in Oncology, Cancer Institute of the State of São Paulo (ICESP), São Paulo 01246-000, Brazil; fabio.marchi@hc.fm.usp.br; 7 Clinical Hospital of the University of Sao Paulo Medical School (HCFMUSP), São Paulo 05403-010, Brazil; 8 Department of Clinical Genetics, University Hospital of Southern Denmark, 7100 Vejle, Denmark; silvia.regina.rogatto@rsyd.dk; 9 Institute of Regional Health Research, University of Southern Denmark, 5230 Odense, Denmark

**Keywords:** *HOXA1*, DNA methylation, oral squamous cell carcinoma, head and neck cancer, tumor suppressor gene

## Abstract

**Simple Summary:**

Head and neck cancer with increased expression levels of *HOXA1* tends to have a worse outcome. This gene is regulated by DNA methylation. Herein, we studied the methylation pattern of *HOXA1* in oral cavity tumors. We found that methyl groups gather more often in a specific region of *HOXA1*, named 3′UTR, in tumor cells compared to healthy tissues. Interestingly, patients with increased methylation levels were preferentially detected in heavier smokers and patients with a longer survival. Our findings suggest that the analysis of the DNA methylation located in the 3′UTR of *HOXA1* could help predict the patient’s prognosis. Our findings could potentially guide treatment decisions and improve patient care in the future.

**Abstract:**

Background: *HOXA1* is a prognostic marker and a potential predictive biomarker for radioresistance in head and neck tumors. Its overexpression has been associated with promoter methylation and a worse prognosis in oral squamous cell carcinoma (OSCC) patients. However, opposite outcomes are also described. The effect of the methylation of this gene on different gene regions, other than the promoter, remains uncertain. We investigated the methylation profile at different genomic regions of *HOXA1* in OSCC and correlated differentially methylated CpG sites with clinicopathological data. Methods: The *HOXA1* DNA methylation status was evaluated by analyzing data from The Cancer Genome Atlas and three Gene Expression Omnibus datasets. Significant differentially methylated CpG sites were considered with a |∆β| ≥ 0.10 and a Bonferroni-corrected *p*-value < 0.01. Differentially methylated CpGs were validated by pyrosequencing using two independent cohorts of 15 and 47 OSCC patients, respectively. Results: Compared to normal tissues, we found significantly higher DNA methylation levels in the 3′UTR region of *HOXA1* in OSCC. Higher methylation levels in tumor samples were positively correlated with smoking habits and patients’ overall survival. Conclusions: Our findings suggest that *HOXA1* gene body methylation is a promising prognostic biomarker for OSCC with potential clinical applications in patient monitoring.

## 1. Introduction

Head and neck cancer stands as the seventh most prevalent cancer worldwide and the cause of death of more than 467,000 individuals [1]. Within this spectrum, oral squamous cell carcinoma (OSCC) emerges as a frequent manifestation, contributing to the head and neck malignancies. A majority of OSCC cases present at advanced stages, with approximately 60% succumbing within five years of diagnosis. Despite advancements, OSCC treatment decisions predominantly rely on TNM classification and clinical staging, although these metrics frequently lack precision in providing prognostic insights [2,3,4]. While surgical resection can be highly effective in early-stage disease, it invariably involves significant functional and structural compromises, particularly regarding mastication and speech [5]. The primary contributor to the high mortality rate associated with OSCC is locoregional recurrence [2].

DNA methylation, an epigenetic mechanism primarily affecting CpG dinucleotides, plays a crucial role in modulating gene expression, which impacts cell differentiation, development, and carcinogenesis [6,7,8,9]. An analysis of DNA methylation profiles offers a valuable understanding of the role of these modifications in both maintaining health and driving disease progression [10]. This epigenetic mark pervades the genome, affecting both gene promoters and bodies. Hypermethylation in the promoters of tumor suppressor genes is a well-established mechanism described in several tumor types, silencing gene transcription by hindering the binding of transcription factors [11]. Conversely, gene body methylation exhibits more nuanced effects, sometimes enhancing gene expression while acting as a subtle regulator in other instances [12,13]. This complex influence extends beyond gene expression, histone modifications, and alternative splicing, and its contribution to both somatic and germline mutations has been increasingly recognized [14,15,16]. This multifaceted and context-dependent nature of gene body methylation has fueled intense research efforts to unravel its impact on cancer development.

The potential of DNA methylation patterns as biomarkers for distinguishing cancer cells from their normal cell counterparts presents exciting opportunities for tailored diagnostic and therapeutic approaches [17,18]. Molecular analyses can offer potential advantages over cell morphology for detecting alterations in normal adjacent tissues (NAT) [19]. Notably, specific molecular changes might be more sensitive than readily discernible cellular morphological changes, potentially enabling the detection of precancerous or early-stage lesions missed by light microscopy [20,21,22,23]. Several studies have investigated the potential of epigenetic changes in normal adjacent margins of the tumor as prognostic factors for OSCC patients [24,25,26,27]. Most of them showed that NAT samples, even if histologically negative for cancer cells, already have changes that can affect diagnosis and prognosis [27,28,29,30].

The methylation profile of tumor suppressor genes has been widely studied in OSCC [11,31,32,33]. Among these genes, *Homeobox A1* (*HOXA1*), located on chromosome 7p15.3 and belonging to the HOX gene family, plays a role in embryonic development, guiding the formation of specific body structures along the body axis, particularly within the head and neck region [34]. An inverse correlation between *HOXA1* expression and its promoter methylation has been predicted [35]. In OSCC patients, elevated *HOXA1* expression levels have been associated with poorer prognoses [35,36], lower levels of CD8+ T cells, and decreased DNA methylation [35]. Nonetheless, the impact of DNA methylation on gene regions beyond the promoter remains unexplored in OSCC. Therefore, our study aimed to investigate the DNA methylation pattern across the *HOXA1* gene in OSCC compared to NAT samples and analyze the associations with relevant clinical parameters.

## 2. Materials and Methods

### 2.1. In Silico Analysis

The present study is a cross-sectional and observational investigation that includes retrospective samples and data. The data analysis was performed using an R v4.1.0 statistical environment. Initially, Infinium HumanMethylation450 Bead Chip (HM450K, Illumina, San Diego, CA, USA) methylation data were downloaded from The Cancer Genome Atlas (TCGA) using UCSC Xena Browser (https://xena.ucsc.edu/, accessed on 23 June 2021). Three datasets were also extracted from the Gene Expression Omnibus (GEO) repository (GSE97784 [37], GSE87053 [11], and GSE75537 [38]).

Patients undergoing surgical treatment for primary OSCC localized on the tongue, floor of the mouth, retromolar area, hard palate, and buccal mucosa were included in the study. The exclusion criteria comprised individuals previously treated for any type of cancer, those with secondary tumors, those with tumor cell percentages below 70%, or those with insufficient clinical data. Additionally, patients lacking annotatable probes, identified as outliers through Multidimensional Scaling Plot analysis, or demonstrating over 10% missing values among probes were excluded. Sample details of the individual cohorts are described in Table 1. Data analyses were conducted using the WateRmelon R package v. 1.34.0 [39].

We filtered probes mapped to regions containing SNPs (Minor Allele Frequency > 5% for all populations according to the 1000 Genomes database) on the X and Y chromosomes and probes that co-hybridize in sequences’ homologous alternatives (≥49 base pairs). Probes were also removed according to the detection of *p* > 0.05 and a bead count < 3 in 5% of the samples. The quality control was performed by distributing the signals obtained for methylated and non-methylated probes and using the Multidimensional Scaling (MDS) analysis. The Beta MIxture Quantile dilation (BMIQ) method was applied to adjust the differences between Type-I and Type-II probes and to normalize the values. Possible batch effects were removed with the sva package, available at Bioconductor. The limma package [40] was used to identify differentially methylated probes (DMPs) between OSCC and NAT samples.

The methylation level of CpG sites was quantified by the β value, which means the proportion of methylated probes for a specific CpG site. This value is calculated by the intensity of the methylated probe divided by the sum of the intensity of the methylated and non-methylated probes. The β value ranges from 0 (demethylated CpG site) to 1 (fully methylated CpG site). Probes with Bonferroni-corrected *p*-values < 1% and a methylation difference (Δβ) ≤ −0.10 or ≥0.10 between the tumor and adjacent normal tissues were considered significant.

RNA-seq data from the TCGA cohort were accessed to support the selection of *HOXA1* for subsequent validation by pyrosequencing. We used the TCGABiolinks package [41] to download RNAseq from the same OSCC samples selected for the DNA methylation analysis (Table 1). To normalize and identify differentially expressed genes between OSCC and NAT tissues, we analyzed the raw count data matrix (STAR-aligned) with the DEseq2 package [42], considering an FDR adjusted *p*-value < 0.05 and log2FC ≥ 1.

### 2.2. HOXA1 Analysis by Pyrosequencing

Two patient cohorts collected from the A.C. Camargo Cancer Center (ACCCC) Tumor Biobank were used to validate the in silico findings. The study was approved by the Institutional Review Board of the ACCCC (protocol codes 2699/19 and 1876/14). The first group (Cohort 1) encompassed 30 paired cryopreserved OSCC and NAT samples from 15 OSCC patients. These cases were used to evaluate 3 CpG sites (cg07659054/chr7:27134225, cg18805066/chr7:27134259, and chr7:27134228) mapped on the *HOXA1* gene body. The second cohort (Cohort 2) included 47 OSCC and 34 paired NAT samples. In this sample set, we investigated a larger number of CpGs using distinct primers, samples, and equipment to further evaluate the prognostic potential of *HOXA1* gene body methylation. The analyzed region comprised 12 CpGs, including cg23865240 (chr7:27134109), cg07659054, and cg18805066. The DNA extraction was performed by QIASymphony, using the QIAsymphony DNA Mini kit (Qiagen, CA, USA). The bisulfite conversion on the samples was carried out using the EZ DNA Methylation-LightningTM kit (Zymo Research, Irvine, CA, USA), following the manufacturer’s instructions. After the conversion, 30 ng of DNA was submitted to *HOXA1* target region amplification using the HotStarTaq DNA Polymerase Kit (Qiagen, Santa Clarita, CA, USA) with the primer sequences described in Table 2. The PCR products of cohorts 1 and 2 were analyzed in the pyrosequencer PyroMark Q96 ID (Qiagen) and PyroMark Q24 equipment (Qiagen), respectively. The primer sequences are displayed in Table 2. Positive and negative commercial methylation controls were used in all reactions (Qiagen).

The inclusion and exclusion criteria were consistent with the standards applied to the in silico analysis. Patients’ medical history and sociodemographic information were assessed, and follow-up data were collected for up to 5 years after diagnosis.

### 2.3. Statistical Analysis

The association between clinical-histopathological data and DNA methylation findings was investigated using IBM SPSS v.23.0 (Chicago, IL, USA). Parametric and non-parametric data were analyzed by Student’s *t* test and the Mann–Whitney test, respectively. Pearson or Spearman correlation tests were used to associate quantitative variables. Overall and recurrence-free survival were estimated for selected CpG sites using the Kaplan–Meier method and compared using the log-rank test.

A significance level of 5% was adopted to discriminate significant from non-significant values. An R v.4.1.0 statistical environment (R Foundation for Statistical Computing, Vienna, Austria) was used to generate graphical representations of the results.

## 3. Results

### 3.1. Differentially Methylated Sites between OSCC and NAT Tissues

**Figure 1:**
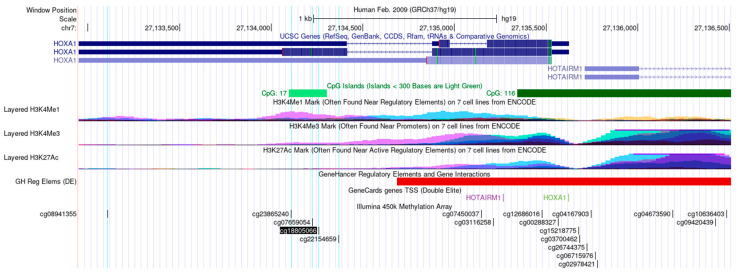
Genomic location provided by the UCSC Genome Browser for the 18 *HOXA1* CpG sites evaluated by the HM450K array. Five significant DMPs from TCGA are highlighted in light blue. Additional genomic characteristics, including CpG islands, histone marks, and transcription factor binding sites, are depicted.

The HOXA1 DNA methylation using the TCGA dataset (HM450K) revealed 18 CpG sites distributed in different regions (Figure 1). We identified five CpGs hypermethylated in OSCC compared to the adjacent tissues (∆β ≥ 0.10 and adjusted *p*-value < 0.01; Table 3, in bold). These CpGs were in the gene body and 3′ untranslated region (3′UTR) of the HOXA1 gene (Figure 1). Using the same selection criteria adopted for TCGA, we found that all three publicly available OSCC datasets from GEO presented the same CpGs differentially methylated between OSCC and NAT (Table 3, marked with ‘*’).

**Figure 2:**
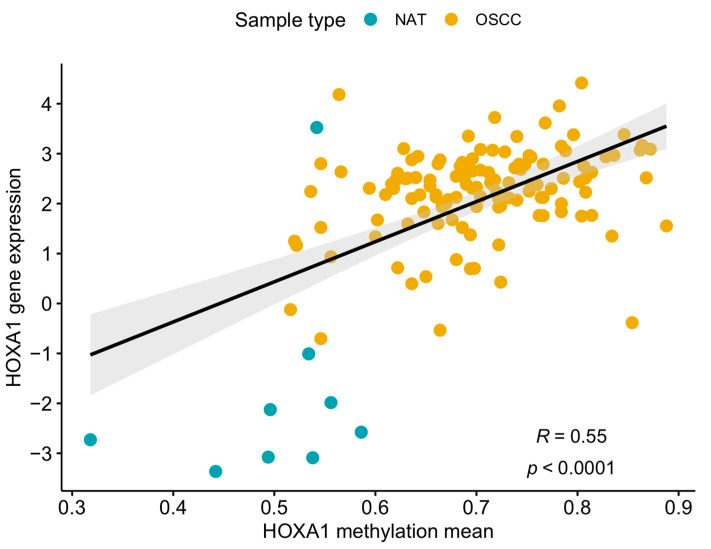
Scatterplot representative of the relationship between HOXA1 methylation and expression levels in the TCGA-OSCC dataset. Blue dots represent normal adjacent tissue (NAT) samples; yellow dots represent oral squamous cell carcinoma (OSCC) tumor samples. The regression line’s confidence interval is represented in light gray.

We assessed the transcriptome data (RNA-Seq) of 136 samples available on TCGA (9 NAT and 127 OSCC) to identify a potential relationship between HOXA1 DNA methylation and gene expression. We detected an increased HOXA1 expression in OSCC samples compared to adjacent tissue samples (fold change = 10.55, *p*-value = 7.1 × 10^−20^) and a positive correlation between DNA methylation and gene expression levels (Figure 2). These findings suggested that methylation in these regions could increase gene expression.

### 3.2. Validation of the DMPs

Two independent cohorts comprising 15 paired OSCC and NAT samples (cohort 1) and 34 paired cases plus 13 unpaired OSCC samples (cohort 2) were evaluated by pyrosequencing, which was performed using different primer sets and sequencers. For both assays, the methylation percentages among tumor and normal samples were compared.

**Figure 3:**
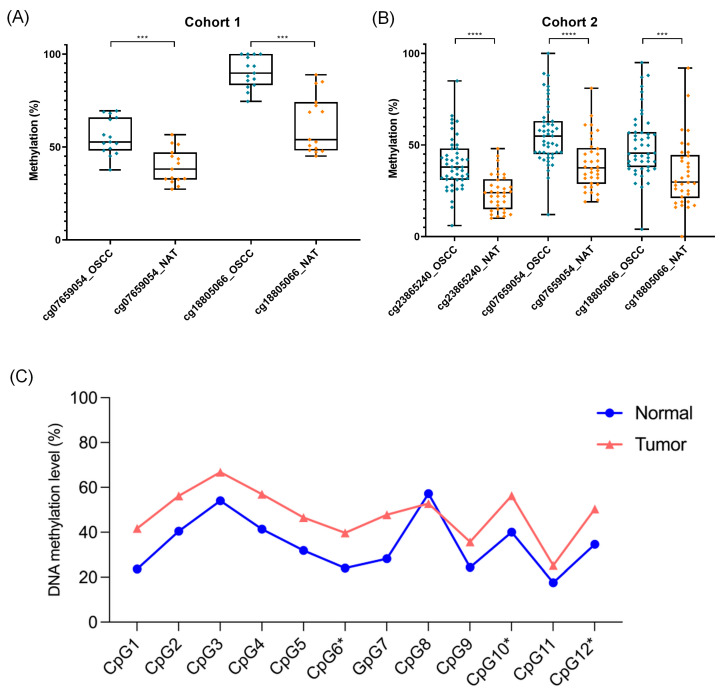
CpG methylation levels between OSCC and normal adjacent tissue (NAT) in the validation groups: (**A**) cohort 1 and (**B**) cohort 2. ***, *p* < 0.001; ****, *p* < 0.0001. (**C**) *HOXA1* methylation pattern evaluated in 12 CpG sites in tumor (OSCC) and normal (NAT) samples. Wilcoxon matched pairs signed rank test, *p* = 0.001. CpG6* = cg23865240, CpG10* = cg07659054, CpG12* = cg18805066.

Two differentially methylated CpG sites (cg07659054 and cg18805066) found on the in silico analysis were validated using the cohort 1 samples (Figure 3A). Recognizing the potential for broader methylation-mediated effects, we sought to clarify the influence of methylation alterations on additional CpG dinucleotides within the HOXA1 region, beyond the limited scope of the initial validation cohort. Thus, in cohort 2, we observed that, except for CpG8, the OSCC samples displayed increased methylation levels of HOXA1 compared to the NAT samples, including the three specific CpGs previously identified by the in silico evaluation (cg23865240, cg07659054, and cg18805066; Figure 3B,C). Those three CpG sites exhibited similar methylation patterns compared to the previously published datasets, with a ∆β value between OSCC and NAT samples of 15.6% for cg23865240, around 16.0% for cg07659054, and ranging from 15.8 to 27.8% for cg18805066 (Table 4).

### 3.3. Association of HOXA1 Methylation with Clinicopathological Data

The median age at diagnosis was 57 years. In both cohorts, most of the patients were male and had lesions in the tongue, and an advanced tumor stage was frequently detected (III/IV). During the study period, up to 40% of the patients presented recurrence. The clinical data of the patients are included in Table 5.

**Figure 4:**
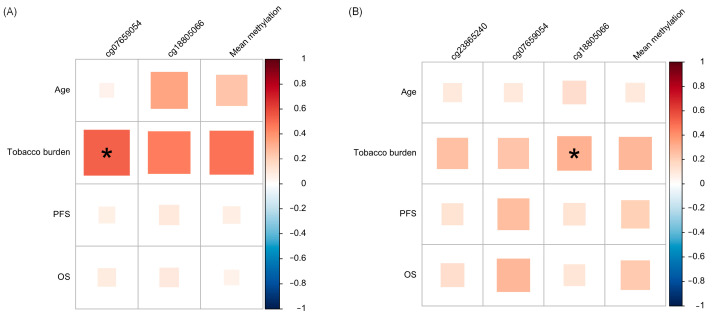
Correlation matrix between *HOXA1* DNA methylation in OSCC and NAT samples and age and smoking habits in (**A**) cohort 1 and (**B**) cohort 2. * Significant associations. OS: Overall Survival. PFS: Progression-free survival.

For each validation group, the average of the methylation levels of the significant DMPs on OSCC samples was calculated and then compared with clinicopathological data, in addition to the three validated CpG comparisons. The OSCC samples’ methylation profiles of cg07659054 in cohort 1 and cg18805066 in cohort 2 were positively correlated with the patients’ smoking habits (r = 0.530, *p* = 0.042 and r = 0.308, *p* = 0.042, respectively; Figure 4A,B).

**Figure 5:**
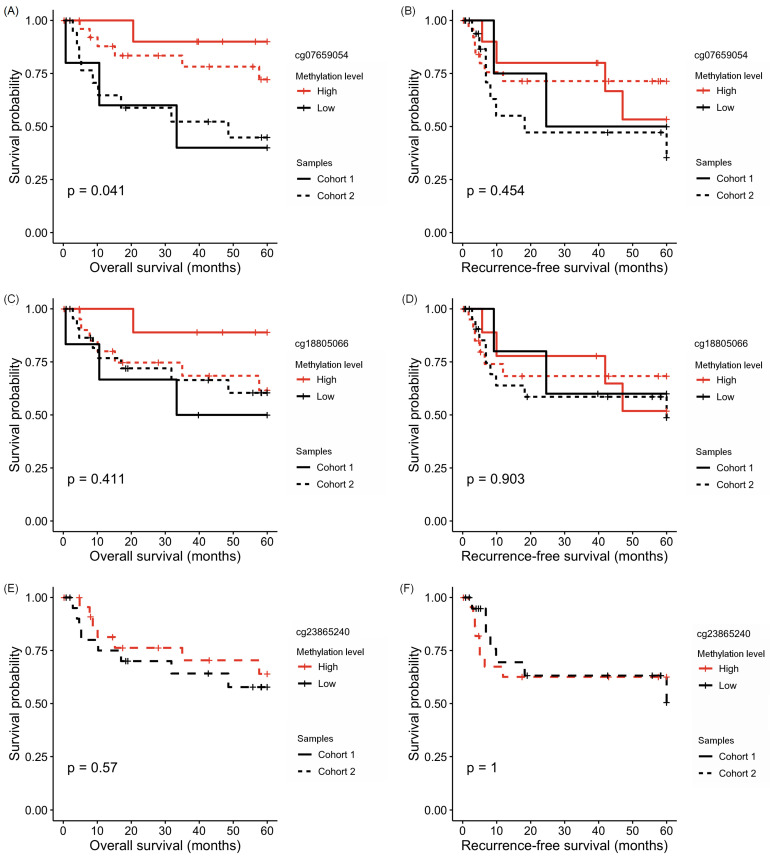
*HOXA1* methylation prognostic potential in the validation cohorts. Kaplan–Meier curves of OSCC patients according to the high and low DNA methylation levels of (**A**) cg0765905 for overall survival (OS), (**B**) cg0765905 for relapse-free survival (RFS), (**C**) cg18805066 for OS, (**D**) cg18805066 for RFS, (**E**) cg23865240 for OS, and (**F**) cg23865240 for RFS. The solid line represents patients included in cohort 1, while the dashed line represents patients from cohort 2.

The methylation of OSCC tissues was further associated with 5-year overall or disease-free survival (Figure 5). Patients with higher levels of the methylation of cg0765905 in tumor samples presented a prolonged overall survival (OS) compared to patients with lower levels of the methylation of this *HOXA1* CpG position (Figure 5A). The other CpG sites were unrelated to OS or relapse-free survival (RFS; Figure 5B–F). No additional association was found between *HOXA1* methylation and the patients’ clinicopathological data.

## 4. Discussion

Highly methylated promoter regions of several tumor suppressor genes have been reported in OSCC. However, few studies have evaluated DNA methylation in other gene regions and outside of CpG islands. In this study, we aimed to characterize the *HOXA1* methylation profile across different genomic locations to elucidate the molecular factors that influence OSCC patient prognosis. We identified and validated the hypermethylated region of the *HOXA1* gene body in OSCC samples compared to normal samples. The increased methylation levels were positively associated with the tobacco consumption and overall survival of the patients.

Despite the gap of knowledge in this area, McGuire et al. [43] investigated, in 10 types of tumors, the relationship between the methylation of the 3′UTR and gene expression. The authors identified that the DNA methylation of this region and gene expression are positively correlated, suggesting that methylation in these regions can increase the transcription rate. In this study, we investigated 18 CpG sites along the *HOXA1* gene and found five differentially methylated sites between OSCC and adjacent normal tissue, all of them located in the 3′UTR and body of the gene and with hypermethylation in tumor samples. Increased methylation in the gene body is believed to be present in actively transcribed genes. According to Jones [44], methylation in the gene body contributes to carcinogenesis, causing mutations in somatic and germ cells, and is positively correlated with gene expression levels.

*HOXA1* encodes a DNA-binding transcription factor that can regulate cell expression, morphogenesis, and differentiation. Studies in OSCC and head and neck cancers reported increased expression levels of this gene [36,45,46]. Mohanta et al. [45] found an aberrant expression of this gene in cancer stem cells and an association with tumor recurrence. Contrasting this evidence, other studies reported that *HOXA1* downregulation is associated with a shorter survival rate [47], and its elevated expression could hinder tumor progression and enhance anti-tumor immune responses by reducing the immunosuppressive activity of myeloid-derived suppressor cells in lung cancer [48]. The present study is the first to report hypermethylation in the 3′UTR region of *HOXA1*, which may be directly correlated with increased expression in oral cancer. Other studies have shown the hypermethylation of *HOXA1* in cholangiocarcinoma [49] and thyroid carcinoma [50].

The analysis of three *HOXA1* CpG sites using the OSCC-TCGA and independent OSCC cohorts revealed significant hypermethylation in OSCC compared to NAT. As previously mentioned, methylation in 3′UTR can positively control gene expression, resulting in *HOXA1* aberrant expression in cancer [25,34,42,43]. Furthermore, the data from OSCC-TCGA showed increased expression levels of *HOXA1* in tumor samples compared to those in adjacent normal samples. We suggest that the hypermethylation of the 3′UTR region in OSCC samples is related to increased gene expression. This association needs further investigation since *HOXA1* gene expression was not investigated in our study. A larger number of cases should be evaluated to confirm the association between increased hypermethylation in OSCC samples and improved overall survival.

The influence of smoking as a cause of methylation in tumor suppressor genes in different tumor types has been described [51,52]. Alexandrov et al. [53] found differentially methylated CpGs in smokers with lung adenocarcinoma and oral cancer when compared to non-smokers; however, these sites did not belong to genes involved in carcinogenesis. Nevertheless, to our knowledge, there are no studies that have evaluated the association between smoking habits and the methylation of the *HOXA1* 3′UTR region in the literature. Thus, further investigation of this possible causality is suggested.

Herein, we used two distinct cohorts with different methylation analysis methods for validating *HOXA1* DMPs, increasing the sample size (*n* = 47) but introducing limitations. The smaller sample size in cohort 1 (*n* = 15) reduced the statistical power, potentially impacting the reliability of our conclusions. However, combining data from two cohorts was not feasible due to technical limitations and differences in the sample collection. Moreover, it is well established that the area surrounding a tumor, even if microscopically normal, often harbors molecular alterations due to field cancerization extending beyond the visible margin [19,54]. Using such tissue as a control can lead to underestimating the differences between normal and tumor samples. NAT offers a readily available alternative, reducing the need for additional interventions. Although NAT displays molecular and cellular changes suggestive of pre-cancerous or field cancerization features, these changes represent an intermediate state between healthy and tumor tissues [55]. Therefore, despite methodological differences, NAT is a valuable control for relative comparisons within the same study, as demonstrated by our findings. Future studies with a larger cohort integrating DNA methylation and gene expression analysis are warranted. This would strengthen the evidence supporting the observed *HOXA1* epigenetic alterations and their potential role in OSCC.

## 5. Conclusions

In summary, we demonstrated that *HOXA1* is generally highly methylated in the gene body and 3′UTR regions, which may have an adverse impact on transcript levels. We observed a positive correlation between smoking habits and higher methylation levels of *HOXA1* CpG sites located at the 3’UTR region. *HOXA1* 3′UTR CpG sites can potentially control and induce increased gene expression. 

## Figures and Tables

**Table 1:** Number of samples included in the datasets used in the exploratory (in silico) analysis.

Cohort	No. of Samples		
	OSCC	NAT	Total
TCGA	127	9	136
GSE75537	54	29	83
GSE97784	12	12	24
GSE87053	11	10	21

OSCC: Oral Squamous Cell Carcinoma; NAT: normal adjacent tissue; TCGA: The Cancer Genome Atlas.

**Table 2:** Primer sequences for the *HOXA1* validation by pyrosequencing.

	Forward (5′-3′)	Reverse (5′-3′)	Sequencing (5′-3′)
Cohort 1	TAGTGGGAGGTAGTTAGAGTG	[Btn]CCAACTTCACTACCAAACAAC	TTTTGTTTTATT
Cohort 2	AGGTAGTTAGAGTGTTTGAGGTAGAA	[Btn]AAAAAATTCCACTTCAACAAATACCT	AGTGTTTGAGGTAGAAG

[Btn]: Biotin Oligonucleotide Modification.

**Table 3:** Differential methylation between OSCC and NAT samples in the 18 *HOXA1* CpG sites in the TCGA and GEO datasets. The five hypermethylated DMPs in OSCC compared to NAT from the TCGA dataset are indicated in bold.

CpG ID	Location	TCGA		GSE75537		GSE97784		GSE87053	
		∆β	adj. *p*-val	∆β	adj. *p*-val	∆β	adj. *p*-val	∆β	adj. *p*-val
**cg18805066**	3′UTR; Body	0.32	1.04 × 10^−38^	0.14	6.73 × 10^−7^ *	0.20	2.28 × 10^−3^ *	0.13	1.15 × 10^−2^
**cg07659054**	3′UTR; Body	0.24	3.46 × 10^−31^	0.11	5.36 × 10^−6^ *	0.17	3.91 × 10^−3^ *	0.13	2.44 × 10^−3^ *
**cg23865240**	3′UTR; Body	0.24	3.11 × 10^−33^	0.12	1.13 × 10^−7^ *	0.19	2.00 × 10^−3^ *	0.14	2.89 × 10^−3^ *
**cg22154659**	3′UTR; Body	0.23	5.13 × 10^−36^	0.08	1.08 × 10^−4^	0.16	4.96 × 10^−3^ *	0.08	6.35 × 10^−2^
**cg08941355**	3′UTR	0.11	1.06 × 10^−11^	0.06	1.88 × 10^−3^	0.15	4.55 × 10^−3^ *	0.11	4.78 × 10^−3^ *
cg10636403	TSS1500	0.02	2.27 × 10^−1^	0.01	5.92 × 10^−1^	0.03	3.14 × 10^−2^	0.01	3.11 × 10^−1^
cg06715976	TSS200	0.02	2.99 × 10^−1^	0.02	2.49 × 10^−1^	−0.01	7.52 × 10^−1^	−0.01	3.34 × 10^−1^
cg02978421	TSS200	0.01	2.92 × 10^−1^	0.02	2.29 × 10^−1^	0.00	7.07 × 10^−1^	0.00	9.27 × 10^−1^
cg04673590	TSS1500	0.01	4.14 × 10^−1^	0.01	4.41 × 10^−1^	0.00	8.16 × 10^−1^	0.00	9.05 × 10^−1^
cg15218775	TSS200	0.01	5.81 × 10^−1^	0.02	2.01 × 10^−1^	−0.01	5.21 × 10^−1^	0.00	8.38 × 10^−1^
cg12686016	1stExon	0.00	9.92 × 10^−1^	−0.01	5.21 × 10^−1^	−0.02	8.56 × 10^−2^	−0.02	2.52 × 10^−1^
cg04167903	TSS200	0.00	9.40 × 10^−1^	0.00	8.30 × 10^−1^	−0.01	4.05 × 10^−1^	0.00	7.72 × 10^−1^
cg07450037	Body; 1stExon	0.00	9.43 × 10^−1^	−0.08	1.33 × 10^−3^	−0.06	1.61 × 10^−1^	−0.07	2.67 × 10^−1^
cg09420439	TSS1500	0.00	8.22 × 10^−1^	0.00	9.22 × 10^−1^	−0.01	1.79 × 10^−1^	−0.02	9.49 × 10^−2^
cg00288327	5′UTR; 1stExon	0.00	7.38 × 10^−1^	−0.01	7.00 × 10^−1^	−0.01	3.66 × 10^−2^	−0.01	3.46 × 10^−1^
cg03700462	TSS200	−0.01	5.97 × 10^−1^	0.01	5.32 × 10^−1^	0.00	7.19 × 10^−1^	−0.01	1.18 × 10^−1^
cg26744375	TSS200	−0.01	4.67 × 10^−1^	0.01	6.87 × 10^−1^	−0.01	2.31 × 10^−1^	−0.01	1.67 × 10^−1^
cg03116258	1stExon	−0.07	2.21 × 10^−4^	−0.10	1.19 × 10^−8^	−0.07	7.91 × 10^−3^	−0.09	1.33 × 10^−3^

∆β: delta-beta; TCGA: The Cancer Genome Atlas; adj. *p*-val: Bonferroni adjusted *p*-value. * Significant probes that followed the same selection criteria for each independent cohort (∆β ≥ 0.10 and adjusted *p*-value < 0.01).

**Table 4:** Methylation levels obtained by pyrosequencing for the validated CpG sites, according to investigated tissue.

CpG ID	Sample	Cohort 1			Cohort 2		
		Mean ± SD	Δβ (%)	*p* *	Mean ± SD	Δβ (%)	*p* *
cg23865240	OSCC	NA	NA	NA	39.6 ± 14.5	15.6	<0.001
	NAT	NA			24.0 ± 9.9		
cg07659054	OSCC	55.5 ± 9.9	15.9	<0.001	56.1 ± 16.7	16.0	<0.001
	NAT	39.6 ± 9.3			40.1 ± 14.4		
cg18805066	OSCC	90.3 ± 8.4	27.8	<0.001	50.3 ± 17.8	15.8	<0.001
	NAT	62.5 ± 15.7			34.5 ± 18.4		

SD: standard deviation. *: *t* Student’s paired test. NA: not available.

**Table 5:** Clinicopathological data from patients included in the validation groups.

Characteristic	Category	Cohort 1 *n* = 15 (%)	Cohort 2 *n* = 47 (%)
Sex	Male	11 (73.3)	35 (76.1)
	Female	4 (26.7)	11 (23.9)
Tobacco consumption	No	7 (46.7)	13 (28.3)
	Yes	6 (40.0)	24 (52.2)
	Former (>2 years)	2 (13.3)	9 (19.6)
Alcohol consumption	No	6 (40.0)	8 (17.4)
	Yes/social	7 (46.7)	33 (71.8)
	Former (>2 years)	2 (13.3)	5 (10.9)
Anatomic site	Tongue	12 (80.0)	24 (52.2)
	Floor of the mouth	3 (20.0)	6 (13.0)
	Gingiva	0 (0.0)	9 (19.6)
	Other	0 (0.0)	7 (15.2)
Clinical staging	I/II	6 (40.0)	13 (28.9)
	III/IV	9 (60.0)	32 (71.1)
Recurrence	No	9 (60.0)	29 (65.9)
	Yes	6 (40.0)	15 (34.1)
Patient status	Alive	11 (73.3)	29 (63.0)
	Dead	4 (26.7)	17 (36.9)

## Data Availability

The data supporting this study’s findings are available from the corresponding author, upon reasonable request.
